# Novel Benzenesulfonamide Derivatives of 5′-Aminospirotriazolotriazine Exhibit Anti-Inflammatory Activity by Suppressing Pro-Inflammatory Mediators: In Vitro and In Vivo Evaluation Using a Rat Model of Carrageenan-Induced Paw Edema

**DOI:** 10.3390/biomedicines13071732

**Published:** 2025-07-15

**Authors:** Amany M. Hamed, Souhaila S. Enaili, Walaa I. Mohammed, Azza M. A. Abouelella, Zeyad Elsayed Eldeeb Mohana, Dina M. Monir, Safaa S. Soliman, Elsayed Eldeeb Mehana Hamouda, Hytham Mahmoud Abd Elatif, Ahmed M. El-Saghier

**Affiliations:** 1 Chemistry Department, Faculty of Science, Sohag University, Sohag 82524, Egypt; el.saghier@science.sohag.edu.eg; 2 Pharmaceutical Chemistry Department, Faculty of Pharmacy, University of Zawia, Al Zawia 16418, Libya; s.enaili@zu.edu.ly; 3 Department of Clinical Pharmacology, Faculty of Medicine, Sohag University, Sohag 82524, Egypt; walaa_mohamed@med.sohag.edu.eg (W.I.M.); azza_abouelella@med.sohag.edu.eg (A.M.A.A.); haitham.mahmoud@bcu.ac.uk (H.M.A.E.); 4 College of Medicine, Alexandria University, Alexandria 26571, Egypt; zeyadhamouda@gmail.com; 5 Physiology Department, Faculty of Medicine, Sohag University, Sohag 82524, Egypt; dina_mounier@med.sohag.edu.eg; 6 Department of Zoology, Faculty of Science, Minia University, Minya 61519, Egypt; safaa.sayed@mu.edu.eg; 7 Department of Pathology, College of Veterinary Medicine, Alexandria University, Alexandria 26571, Egypt; elsayedhamouda@gmail.com; 8 Department of Basic Medical Science, Al Rayan National College of Medicine, Madinah 41411, Saudi Arabia

**Keywords:** 1,3,5-triazine derivatives, anti-inflammatory, carrageenan, MTT assay, cox isoenzymes, indomethacin

## Abstract

**Background/Objectives**: Inflammation is a crucial and complex mechanism that protects the body against infections. In our study, we propose to provide scientific evidence for the anti-inflammatory properties of 1,3,5-triazine derivatives. **Methods**: Initially, we ensured the safety of the three synthesized derivatives by administering graded doses of up to 2000 mg/kg intraperitoneally in Wistar rats. Thus, the three derivatives were considered generally safe. We also evaluated their ability to reduce carrageenan-induced rat paw edema. **Results**: Compounds **1**, **2**, and **3** demonstrated stronger anti-inflammatory activity than indomethacin (10 mg/kg), achieving maximum inhibition at the fourth hour with percentages of 96.31%, 72.08%, and 99.69%, respectively, at a dose of 200 mg/kg, compared to 57.66% for the standard drug. To explore the mechanism, levels of pro-inflammatory cytokines (TNF-α, IL-1α, IL-1β, IL-6, CRP) and oxidative stress markers were measured in paw tissue. All three compounds significantly reduced these markers more effectively than indomethacin and enhanced antioxidant levels (SOD and GSH) beyond those achieved by the standard treatment. Additionally, the compounds reduced COX-1 and COX-2 levels to values comparable to those in the normal (non-inflamed) control group. **Conclusions**: Compounds **1**, **2**, and **3** at doses of 200 mg/kg significantly (*p*  < 0.05) inhibited the heat-induced hemolysis of red blood cell (RBC) membranes by 94.6%, 93.9%, and 95.2%, respectively, compared to 94.5% produced by indomethacin. Consequently, we concluded that 1,3,5-triazine derivatives are a safe antioxidant agent with significant anti-inflammatory activity.

## 1. Introduction

The most often used medications for treating various inflammations are non-steroidal anti-inflammatory drugs (NSAIDs), which inhibit cyclooxygenase enzymes [1]. Common NSAIDs block both the COX-1 and COX-2 isoenzymes, which is mostly the reason for the adverse effects on the gastrointestinal tract. Lowering prostaglandins and thromboxanes, which cause inflammatory effects, results from inhibiting the COX-2 isoenzyme [2]. Therefore, a selective COX-2 inhibitor is always needed to have an anti-inflammatory medication without any gastrointestinal side effects [3]. Medications like celecoxib, valdecoxib, and rofecoxib are the most significant medications that are markedly selective COX-2 inhibitors [4]. However, there are certain adverse effects related to myocardial thrombosis associated with their administration. Finding novel anti-inflammatory drugs with the potential for clinical application and no or few side effects is therefore a key objective in medicinal chemistry [5,6]. Recently, the technique of combining two or more pharmacophores into a single molecule has been widely applied in drug design and discovery to bring new anti-inflammatory drugs with strong action and reduced adverse effects [7,8]. A wide range of biological actions, including antifungal, anti-HIV, anticancer, anti-inflammatory, analgesic, and antihypertensive, have been discovered for triazine derivatives [9].

**Figure 1:**
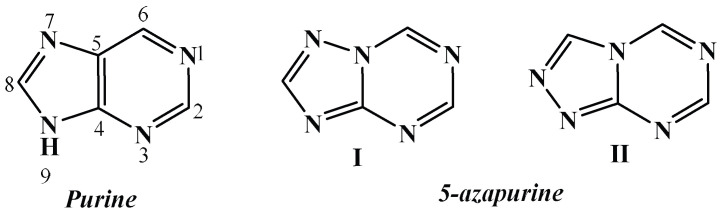
Purine and triazolotriazine isomers’ structures.

Purine bio-isostere is hence the subject of much investigation and usage by pharmaceutical chemists in the development of novel pharmacological substances. Both [1,2,4]triazolo[4,3-a][1,3,5]triazine (I) and [1,2,4]triazolo[1,5-a][1,3,5]triazine (II) (Figure 1) are variants of the heterocycle ring of 1,3,5-triazine, which functions as a bio-isostere of purine and has been extensively studied as an adenosine receptor blocker [10,11]. These isoforms depend on the locations of the nitrogen atoms and the variety of derivatives that may be created from them [12]. Because it selectively inhibits cyclooxygenase (COX-2), the 1,3,5-triazine ring serves as a crucial pharmacophore with analgesic and anti-inflammatory properties [13,14].

Lysosomal enzymes are released when inflammation occurs, and these enzymes are responsible for a variety of diseases that cause tissue damage by damaging macromolecules and causing lipid peroxidation in membranes. These enzymes have been shown to have extracellular activity associated with either acute or chronic inflammation, and lysosomal membrane stabilization plays a crucial role in regulating the inflammatory response. In response, lysosomal components of active neutrophils, such as proteases and bactericidal enzymes, are prevented from being released, which leads to further tissue inflammation and injury [15].

Inflammation is a complex process that is typically associated with pain and includes processes like increased vascular permeability, increased protein denaturation, and membrane alterations. The red blood cell (RBC) or erythrocyte membrane is analogous to the lysosomal membrane. Its stabilization suggests that the synthesized chemicals may have a similar effect on lysosomal membranes. The stability of the RBC membrane is widely used as an in vitro marker of an anti-inflammatory drug’s efficacy [16].

Because of the biological effects of each system and their reciprocal interaction between the two heterocyclic rings [17,18,19], the hybridization of 1,2,4-triazole and 1,3,5-triazine in a single molecule may produce new compounds with enhanced anti-inflammatory agents [20,21,22].

The growing interest in drug development and pharmacological research has led to increased synthesis of 1,3,5-triazine derivatives and evaluation of their anti-inflammatory potential. In many studies, indomethacin has been used as a standard reference drug for comparison.

However, building upon our previous work, we have previously synthesized and characterized a series of spirotriazolotriazine derivatives, among which compounds **7**, **8**, and **12** exhibited outstanding in vitro anti-inflammatory activity, as confirmed by RBC membrane stabilization, COX inhibition assays, and molecular docking studies [23]. These compounds demonstrated superior activity even compared to indomethacin, highlighting their potential as promising anti-inflammatory agents. Given their remarkable efficacy and selectivity in preliminary in vitro evaluations, the present study was designed to further explore their biological impact through a more comprehensive assessment, including extended in vitro profiling and in vivo validation, to better understand their mechanism of action and therapeutic potential.

## 2. Materials and Methods

### 2.1. Chemistry

The Fisher-John mechanical method was used to determine the melting points of every synthetic chemical. Sigma-Aldrich was the source of the chemicals and solvents. The KBr technique was used to evaluate the produced compounds’ infrared (ν cm^−1^) spectra. ^1^H NMR (DMSO-*d*_6_) (δ ppm) and ^13^C NMR spectra were recorded at Sohag University at 400 MHz and 100 MHz, respectively. The Bruker ADVANCE 400 MHz spectrometer (Bruker BioSpin GmbH, Rheinstetten, Germany) provided the spectra. Elements were analyzed using a Perkin-Elmer CHN analyzer (PerkinElmer Inc., Waltham, MA, USA).

#### General Procedures for the Synthesis of 5′-Amino-N-(4-sulfamoylphenyl)-spiro[1,7′-[1,2,4]triazolo[1,5-a][1,3,5]triazine]-2′-carboxamide Derivatives (**1**–**3**)

A mixture of an appropriate mono- or di-ketone (25 mmol), cyanoguanidine 2 (20 mmol), and 2-hydrazinyl-N-(4-sulfamoylphenyl)-2-thioxoacetamide (1) (20 mmol) was dissolved in ethanol (15 mL) containing concentrated HCl (1 mL). The reaction mixture was heated under reflux with stirring for 2–5 h, depending on the nature of the ketone (reaction progress was monitored by TLC). After completion, the reaction mixture was cooled to room temperature, and the resulting precipitate was filtered, washed with cold ethanol, and dried to afford the corresponding product as crystals.

**5′-Amino-*N*-(4-sulfamoylphenyl). -1′H-spiro[cycloheptane-1,7′-[1,2,4] triazolo[1,5-a][1,3,5] -triazine] -2′-carboxamide (1):** 95% yield, yellow solid. Mp. 180–182 °C. 3382, 3340, 3273, 3181 (2NH, 2NH_2_), 3110. (CHar-om.), 2928, 2749 (CH_aliph_.), 1693 (C=O_amidic_, st), 1664 (C=N), and 1161 (S=O, st) are the FT IR (KBr) maximum cm^−1^. 1H-NMR (DMSO-*d*6), δ ppm: 11.34 (s, 1H, NH_amide_), 10.39 (s, 1H, NH_triazine_), 8.01 (s, 2H, NH_2triazine_), 7.87–7.71 (m, 4H, CH_arom_.), 7.73 (s, 2H, NH_2_), 2.05–1.62 (m, 12H, 6CH_2cycloheptane_); ^13^C-NMR (DMSO-*d*6), δ ppm: 156.09 (C=O_amidic_), 156.00 (C_triazine_), 154.93 (C_triazole_), 152.63 (C=N), 141.53, 137.00 (2C_arom_.), 126.90, 120.07 (2CH_arom_.), 90.63 (C_spiro_), 34.67, 28.13, and 22.88 (6CH_2cycloheptane_); C.F. (M.W.): C_17_H_22_N_8_O_3_S (418.48); Elemental Analysis: C, 48.79/48.82; H, 5.30/5.28; N, 26.78/26.76; S, 7.66/7.65% are the calculated/found values.

**5′-Amino-3,3-dimethyl-5-oxo-*N*-(4-sulfamoylphenyl). -1′H-spiro[cyclohexane-1,7′-[1,2,4]. triazolo[1,5-a]. [1,3,5]triazine -2′-carboxamide (2):** 0.91% yield, white–yellow. FT IR (KBr) Mp. 270–272 °C: max cm^−1^: 3377, 3326, 3278, 3278, 3242, 3180 (2NH, 2NH_2_), 3104, 3054 (CH_arom_.), 2932, 2856 (CH_aliph_.), 1725 (C=O_cyclic_), 1692 (C=O_amide_, st), 1631 (C=N), and 1161 (S=O, st). ^1^H-NMR (DMSO-*d_6_*), δ ppm: 11.34 (s, 1H, NH_amide_), 10.49 (s, 1H, NH_triazine_) 8.30 (s, 2H, NH_2triazine_), 8.01–7.92 (m, 4H, CH_arom_.), 7.82 (s, 2H, NH_2_), 4.85–4.75 (m, 2H, CH_2_), 4.12–3.60 (m, 4H, 2CH_2_), 1.19–1.04 (m, 6H, 2CH_3_); ^13^C-NMR (DMSO-*d_6_*), δ ppm: 215.66 (C=O), 167.95 (C=O_amidic_), 159.11 (C_triazine_), 157.13 (C_triazole_), 155.30 (C=N), 141.04, 140.19 (2C_arom_.), 127.05, 120.15 (2CH_arom_.), 90.54 (C_spiro_), 61.09 (CH_2_-C=O), 42.07 (2CH_2_), 20.27 (2CH_3_), and 14.63 (C-(CH_3_)_2_); C.F(M.W.); C_18_H_22_N_8_O_4_S (446.15); elemental analysis: calc./found: C, 48.42/48.44; H, 4.97/4.95; N, 25.10/25.06; S, 7.18/7.15%.

**5′-Amino-*N*-(4-sulfamoylphenyl) -1′H,10H-spiro [anthracene-9,7′-[1,2,4] triazolo[1,5-a] [1,3,5]triazine -2′-carboxamide (3)**: Pale orange solid, yield (93%). Mp. 172–174 °C. ^13^C-NMR (DMSO-*d*_6_), δ ppm: 158.83 (C=O_amidic_), 149.31 (C_triazine_), 141.53 (C_triazole_), 140.53 (C=N), 135.03, 133.51 (2C_arom_.), 132.42, 131.71 (2C_arom_.), 129.59, 128.36 (2C_arom_.), 127.06, 126.03, 124.13, 123.53, 120.47, 117.29 (6CH_arom_.), 85.80 (C_spiro_) and 31.80 (CH_2_.); FT IR (KBr) ν max cm^−1^: 3381, 3334, 3334, 3277, 3179 (2NH, 2NH_2_), 3070 (CH_arom_.), 2925, 2873 (CH_aliph_.), 1694 (C=O_amide_, st), 1162 (S=O, st), and 1661 (C=N). ^1^H-NMR (DMSO-*d*_6_), δ ppm: 11.30 (s, 1H, NH_amide_), 10.50 (s, 1H, NH_triazine_), 8.44 (s, 2H, NH_2 triaizine_), 8.21–7.51 (m, 12H, CH_arom_.); 7.05 (s, 2H, NH_2_); 4.48 (s, 2H, CH_2_); C.F(M.W.): C_24_H_20_N_8_O_3_S (500.54); C, 57.59/57.61; H, 4.03/3.97; N, 22.39/22.37; S, 6.41/6.39% are the calculated/found elements.

### 2.2. Reagents and Reference Drugs

Carrageenan (CAS No. 9000-07-1) and indomethacin (CAS No. 53-86-1) were supplied by Sigma-Aldrich (St. Louis, MO, USA). The selective pro-inflammatory cytokines were measured quantitatively using rat-specific Enzyme-Linked Immunosorbent Assay (ELISA) kits from Thermo Scientific™ (Waltham, MA, USA) with CAT. No. ERCRP for CRP; from Elabscience with Cat. No. E-EL-R2856 for TNF-α and with Cat. No. E-EL-R0011 for IL-1α; from CUSABIO, Houston, TX, USA, with Cat. No. CSB-E04640r for IL-6; and from My BioSource Co, USA, with Cat. No. MBS825017 for IL-1β, with Cat. No. MBS753621 for rat cyclooxygenase 1, and with Cat. No. MBS266603 for rat cyclooxygenase 2. Meanwhile, an ovine COX inhibitor-screening in vitro assay (item number 760111) was obtained from Cayman Chemical (Ann Arbor, MI, USA). We purchased malondialdehyde (MDA), glutathione (GSH), and superoxide dismutase (SOD) detection kits from Nanjing Jiancheng Bio-Technology Co. Ltd. (Nanjing, China).

### 2.3. Ethical Considerations

The animal study and all experiment techniques have been permitted by the Committee for Scientific Research Ethics (CSRE) of the Faculty of Science, Sohag University, Sohag, Egypt (protocol number CSRE-44-24) in December 2024.

### 2.4. Biological Activity

Due to the limited aqueous solubility of the synthesized 1,3,5-triazine derivatives (Compounds **1**–**3**), all test compounds were formulated using 2% (*v*/*v*) Tween 80 in normal saline as a vehicle. This approach is commonly employed for poorly water-soluble compounds to ensure uniform dispersion and enhance bioavailability without affecting cellular or systemic integrity. For in vitro experiments, compounds were dissolved to the required concentrations and freshly prepared before each assay. For in vivo studies, the compounds were administered intraperitoneally at a dose of 200 mg/kg, with no signs of precipitation observed throughout the study. This formulation was well tolerated by the animals, as confirmed in the preliminary acute toxicity evaluation.

#### 2.4.1. In Vitro Anti-Inflammatory Activity Assays

The protective effects of the synthesized compounds were evaluated using models such as egg albumin and bovine serum albumin denaturation assays, as well as red blood cell membrane stabilization. Although not direct measures of anti-inflammatory potency, these assays are commonly used to indicate potential anti-inflammatory and cytoprotective activity.

##### Bovine Serum Albumin Denaturation Inhibition Assay

A bovine serum albumin assay was conducted using the previously mentioned technique [24]. Synthesized compounds were dissolved in 2% Tween 80 saline solution (*v*/*v*) to create a solution at different doses of 50, 100, 200, 400, 800, and 1000 μg/mL. To prepare the test control sample (0.5 mL), 0.45 mL of 5% *w*/*v* bovine serum albumin was dissolved in 0.05 mL of distilled water. A product control solution was made by mixing 0.45 mL of Tween 80 with 0.50 mL of the test sample. 0.45 mL of bovine serum albumin and 0.50 mL of synthesized compounds made up the 0.5 mL test solution. Additionally, indomethacin was applied as a standard [25]. A pH of 6.3 was adjusted using 1 N HCl. Every solution was heated for 3 min at 60 °C after being kept at 37 °C for 20 min. Samples were then allowed to cool, and their absorbance at 660 nm was measured. Three duplicates of the procedure were carried out, and the percentage inhibition of denaturation of the bovine serum albumin was calculated using the following formula:

%Inhibition = ((Absorbance Control − Absorbance Sample)/(Absorbance control)) × 100.

##### Egg Albumin Protein Denaturation Inhibition Assay

To determine the egg albumin protein denaturation inhibition assay, the previously mentioned approach was used [26]. Egg albumin (0.2 mL), 2.8 mL of phosphate-buffered saline (pH 6.4), and 2 mL of synthesized compound solutions in various concentrations were added to 5 mL of reaction mixture for this experiment. Indomethacin was the standard control, and Tween 80 was the negative control. The reaction solutions were heated to 37 °C for 15 min, then to 70 °C for 5 min. After cooling, the absorbance of the solutions was measured at 660 nm. The experiment was conducted in triplicate, and the percentage inhibition of denaturation of the protein was calculated using the following formula:

%Inhibition = ((Absorbance Control − Absorbance Sample)/(Absorbance control)) × 100.

##### Red Blood Cell Membrane Stabilization Assay

**Principle**

A few diseases are caused by an enzyme that lysosomes produce in response to inflammation. It is believed that either acute or chronic inflammation is connected to the systemic activity of enzymes. NSAIDs exert part of their anti-inflammatory effect by stabilizing lysosomal membranes, which prevents the release of inflammatory mediators such as proteolytic enzymes during cell injury or stress. Given that the membrane of the RBC and the lysosome are similar, a study was performed to quantify the in vitro anti-inflammatory effect by measuring the membrane’s stability using the synthesized compounds [27,28].

**Preparing erythrocyte suspension**

Three milliliters of fresh whole blood were taken from healthy rats, and the blood was centrifuged for ten minutes at 3000 rpm. A volume of standard saline solution (0.9 percent NaCl) equal to that of the supernatant was used to dissolve the red blood pellets. After determining the volume of the dissolved red blood pellets, a 40% *v*/*v* suspension was prepared using an isotonic buffer solution (10 mM sodium phosphate buffer, pH 7.4). The buffer solution was prepared by dissolving 0.2 g of NaH_2_PO_4_, 1.15 g of Na_2_HPO_4_, and 9 g of NaCl in 1 L of distilled water. The experiment utilized red blood cells that had been washed and then resuspended in this buffer to prepare the working cell suspension.

**Hypotonicity induced hemolysis**

The experiment was conducted using certain drugs, as previously mentioned [29]. To make a hypotonic solution, samples of the synthesized compounds were dissolved in distilled water. The hypotonic solution (5 mL) containing graded dosages of the synthesized compounds (100, 200, 400, 600, 800, and 1000 μg/mL) was added to the centrifuge tubes in duplicate pairs (per dose). In addition, an isotonic solution (5 mL) containing graded dosages of the synthesized compounds (100–1000 μg/mL) was added to duplicate pairs (per dose) of centrifuge tubes. Indomethacin served as the standard medication. The control tubes contained 5 mL of indomethacin at 200 μg/mL and 5 mL of the vehicle, which was distilled water. 0.1 mL of the erythrocyte suspension was added to each tube and gently stirred. The solutions were then incubated for one hour at an ambient temperature (37 °C) and centrifuged for three minutes at 1300 g. The absorbance (OD) of the supernatant at 540 nm was used to calculate the hemoglobin concentration using a Spectron (Milton Roy) spectrophotometer. Assuming that all hemolysis generated in the presence of distilled water is 100%, the hemolysis percentage was estimated. The following formula was used to determine how much hemolysis the triazine derivatives inhibited: % inhibition of hemolysis = (1 − ((OD2 − OD1)/(OD3 − OD1))) × 100 [30], where OD1 represents the absorbance of the test sample in an isotonic solution, OD2 represents the absorbance of the test sample in a hypotonic solution, and OD3 represents the absorbance of the control sample in a hypotonic solution.

##### In Vitro COX Inhibition Assay

The test compounds’ ability to suppress COX-1 and COX-2 was evaluated using a colorimetric COX (ovine) inhibitor-screening method (Cayman Chemical, Item Number 760111) [23]. In brief, 150 μL of assay buffer, 10 μL of heme, 10 μL of enzyme (COX-1 or COX-2), and 10 μL of compound (1 mg/mL) make up the reaction mixture. In a final volume of 1 mL, the test compounds were evaluated at doses of 50, 100, and 150 μg/mL. The cyclooxygenase peroxidase component is used in the experiment. By monitoring the appearance of oxidized N, N, N0, and N0-tetramethyl-p-phenylenediamine (TMPD) at 590 nm, the peroxidase activity is measured calorimetrically. A standard medication, termed indomethacin, was utilized. The following formula was used to determine the % COX inhibition: The COX inhibition activity percentage is equal to (1 − T/C ×100), where T is the inhibitor well’s absorbance and C is the starting activity’s absorbance at 100% without the inhibitor well.

##### Cell Viability and Cytotoxicity Evaluation (MTT Assay)

The cell lines were obtained from VACSERA—Holding Company for Biological Products and Vaccines, Cairo, Egypt. A 96-well tissue culture plate was seeded with 1 × 10^5^ cells/mL (Wl-38 cells) from the human lung fibroblast cell line, adding 100 µL per well, and incubated at 37 °C with 5% CO_2_ for 24 h to allow the formation of a confluent monolayer. Following incubation, the growth medium was aspirated, and the cell monolayers were washed twice with wash medium to remove residual serum. Serial two-fold dilutions of the test compound were prepared in RPMI-1640 supplemented with 2% fetal bovine serum. Each dilution (100 µL) was added to the designated wells, while three wells served as negative controls and received only maintenance medium. The plate was incubated at 37 °C for an appropriate duration, during which cellular morphology was monitored for signs of cytotoxicity, including monolayer disruption, cell rounding, shrinkage, or granulation. MTT solution (5 mg/mL in PBS) was prepared, and 20 µL was added to each well. The plate was briefly agitated at 150 rpm for 5 min and then incubated for 4 h at 37 °C to allow viable cells to metabolize MTT into insoluble formazan crystals. After incubation, the supernatant was carefully removed, and 200 µL of DMSO was added to each well to solubilize the formazan. The plates were agitated again at 150 rpm for 5 min, and the absorbance was measured at 560 nm with background subtraction at 620 nm. The resulting optical density values were directly proportional to the number of viable cells [31].

#### 2.4.2. In Vivo Anti-Inflammatory Activity

##### Pilot Study to Assess Acute Toxicity

According to OECD guidelines (Testing of Chemical Number 423), the study was carried out. In this study, mature male Wistar rats in good health were selected. After a 24 h fast, the animals were divided into groups of six. The test compounds were injected intraperitoneally at dosages ranging from 100 mg to 2000 mg per kilogram, dissolved in 2% Tween 80 saline solutions (*v*/*v*). The animals in the control groups were given just the vehicle, which was Tween 80 in 0.9% normal saline. The animals were monitored for signs and symptoms of overt toxicity at 20 min intervals for 48 h following the test compound’s administration at a dose of 2000 mg/kg, which resulted in mortality (LD 50). Thus, 200 mg/kg (ED 50), which is considered one-tenth of the LD 50, was chosen as the anti-inflammatory dose.

##### Experimental Animals and Carrageenan-Induced Paw Edema

Rats’ carrageenan-induced paw edema model was used to assess the novel synthesized drugs’ anti-inflammatory properties [32]. A total of 180–190 g of male Wistar rats were used for the experiments. In the animal house at Sohag University in Sohag, Egypt, every animal was kept in careful laboratory conditions. After being weighed and divided into six groups at random, the animals are housed for a week to reduce stress and allow them to get used to the laboratory environment. The chemicals that were synthesized were administered intraperitoneally (i.p.) to rats at a dosage of 200 mg/kg. The control group received only vehicles. Indomethacin (10 mg/kg) was administered to the positive control group, or reference group. The rats were given a 0.2 mL sub-plantar injection of a 1% (*w*/*v*) suspension of carrageenan (made in PBS) in their right hind paw thirty minutes following the injection of synthesized compounds [33]. A mercury plethysmometer (Ugo Basil, Gemonio, Italy) was used to measure the paw’s volume both before and four hours after the carrageenan injection. The % inhibition of paw edema was calculated by the following formula [34]:

%Inhibition = ((Mean edema of control group − Mean edema of test compound)/(Mean edema of control group)) × 100.

##### Biochemical and Histopathological Examination

At the end of the experiments, the rats were subjected to anesthesia with chloroform and then sacrificed. Blood was obtained via heart puncture for biochemical analysis. Following the animals’ sacrifice, the paws were taken out, decalcified, and stored in a 10% formalin solution. To prepare the tissue samples, they were sliced into thicknesses of 5 µm [35]. Hematoxylin and eosin dyes were used to create the histology slides, which were then examined using a photomicroscope (Meiji Techno^®^, Iruma, Japan) for pathological alterations [36]. The presence of inflammation was documented. Protease/phosphatase inhibitor cocktail in cold phosphate-buffered saline (1:4) (pH 7, 0.01 mol/L) (Cat. No. PPC1010, Sigma-Aldrich) was used to homogenize each rat’s paw piece in a glass homogenizer. After filtering and centrifuging the resultant homogenate for five minutes at 5000× *g*, oxidative stress biomarkers like GSH, SOD, and MDA were measured. The trials were repeated three times [37].

### 2.5. Statistical Analysis

The information was presented as the mean ± SD of the volume difference (mL) between the control and carrageenan-treated paws. One-way analysis of variance (ANOVA) was used to analyze the experimental groups’ data, and a Tukey post hoc test was then performed. *p* < 0.05 was considered significant. The IC_50_ values were calculated using Microsoft Excel by plotting a dose–response curve between compound concentration and cell viability percentage. A linear regression or logarithmic trendline was fitted to the data points, depending on the curve behavior.

## 3. Results

### 3.1. Chemistry

Considering the importance of spirocyclic systems with triazolo-triazine derivatives, we continue our work in heterocyclic synthesis by concentrating on developing some new benzene-sulfonamides that incorporate 5′-aminospirotriazolotriazine with various substitutions at position 7 that may serve as active pharmacophores.

**Scheme 1:**
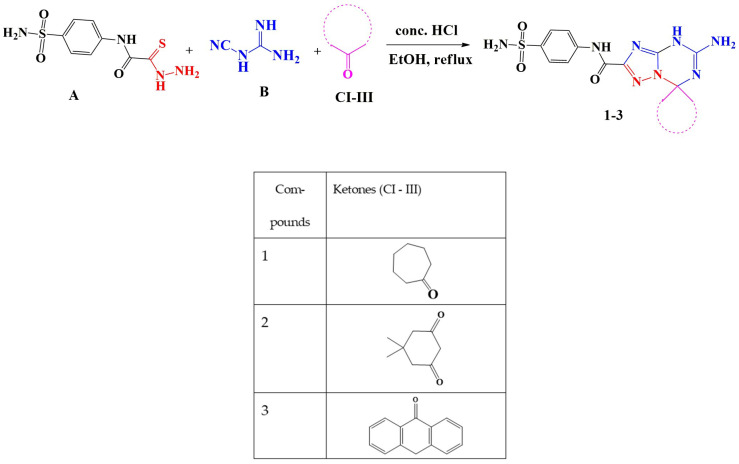
Synthesis of new 5-amino-7-(substituted)-N-(4-sulfamoylphenyl)-4,7-dihydro-[1,2,4]triazolo[1,5-a][1,3,5]triazine-2-carboxamide derivatives (**1**–**3**).

As part of our ongoing research into the synthesis of novel spiro-heterocycles **1**–**3** [38,39,40,41], we synthesized a new series of 5′-aminospirotriazolotriazine derivatives that incorporate benzene and sulfonamide. Cyanoguanidine B and many cyclic and acyclic ketones C_I-III_, including cycloheptanone, dimedone, and anthracen-9(10H)-one, were permitted to react with thiocarbohydrazide A. With stirring and reflux for three hours, the reaction was conducted in ethanol with a few drops of concentrated HCl in a three-component reaction system. Hot precipitation was used to separate products **1**–**3** through filtration (Scheme 1).

**Scheme 2:**
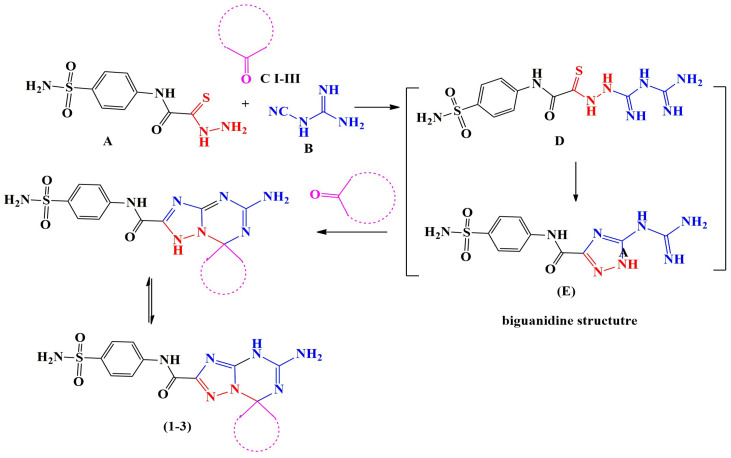
Proposal reaction mechanism for synthesized derivatives of [1,2,4] triazolo [1,5-a][1,3,5] triazine-2’-carboxamide.

In order to produce 5-guanidino-N-(4-sulfamoylphenyl)-1H-1,2,4-triazole-3-carboxamide as an intermediate (E), the reaction mechanism was assumed to be a nucleophilic attack of the amino group of thiocarbohydrazide A at the cyano group of cyanoguanidine B to produce biguanide intermediate (D), followed by a nucleophilic attack of the NH of biguanide at the carbon of thiocarbonyl (C=S) with elimination of H_2_S (as indicated by paper dampened with lead acetate). Furthermore, the required spiro compounds are obtained with the removal of water through nucleophilic attack of the NH (triazole) and NH (guanidine) at the carbonyl group of ketones (Scheme 2).

The physical and spectral data of 1,2,4-triazolo-[1,3,5]triazine derivatives **1**–**3** were verified. The FT-IR spectrum revealed the presence of NH and NH_2_ stretching bands at 3382–3181 cm^−1^, a C=H stretching band at 3070–3110 cm^−1^, a CH_2_ stretching band of cycloalkane at 2932–2749 cm^−1^, and new C=O bands for compound **2** at 1692 and 1725 cm^−1^. Additionally, the absence of the C=S stretching band at 1161 cm^−1^ and the absence of a C-S-C stretching band at 748 cm^−1^ in the FT-IR spectrum provided clear evidence that the reaction had produced the spiro triazolo-triazine system.

The signals in the ^1^HNMR spectra at 11.34–11.30 (s, NH_amide_), 10.50–10.39 (s, NH-_triazine_), 8.44–8.01 (s, NH_2-triazine_), 8.21–7.51 (m, CH-_aromatic_), 7.82–7.05 (s, NH_2_), 4.48 (s, CH_2_-_anthrone_), 4.85–1.62 (m, CH_2_-_cyclic_), and 1.19–1.06 (m, CH_3_-_dimedone_) were all signals that confirmed the formation of compounds **1**–**3**. The synthesis of spiro compounds (**1**–**3**) was shown by a clear band at 91.71–84.90 ppm, and the expected structure of the compounds was confirmed by the aliphatic and aromatic carbon counts in the ^13^C-NMR spectra. Additionally, the elemental analysis spectrum confirmed the compounds’ purity.

### 3.2. Evaluation of Biological Activity

#### 3.2.1. In Vitro Anti-Inflammatory Activities

##### Effect on Protein Denaturation

**Figure 2:**
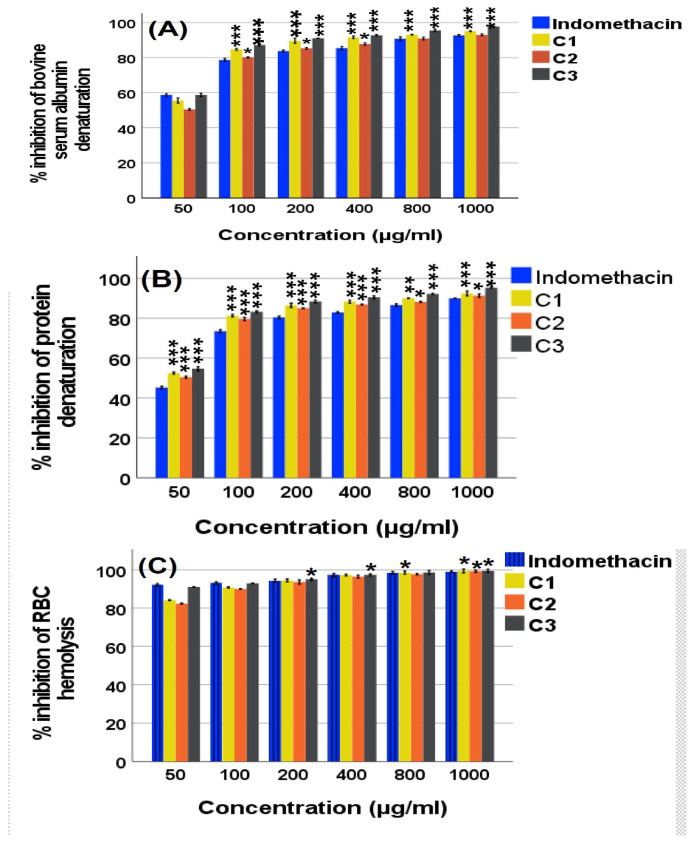
In vitro anti-inflammatory activity of synthesized compounds **1**, **2**, and **3** by using (**A**) bovine serum albumin denaturation, (**B**) protein denaturation, and (**C**) erythrocyte hemolysis methods. All values are expressed as mean ± SD (*n* = 3), using one-way ANOVA followed by Tukey’s posttest. C1, C2, C3: compound **1**–**3**; RBC: red blood cell. * *p* ≤ 0.05 (significant), ** *p* ≤ 0.01 (very significant), *** *p* ≤ 0.001 (highly significant) vs. indomethacin.

Heat-induced denaturation of bovine serum albumin results in the expression of antigens linked to Type III hypersensitivity reaction, which may be indicative of conditions including systemic lupus erythematosus, glomerulonephritis, rheumatoid arthritis, and serum sickness [42]. The anti-inflammatory properties of compounds **1**, **2**, and **3** were investigated using protein denaturation of egg albumin at five different doses (50–1000 µg/mL) and bovine serum albumin (BSA). All tested compounds exhibited a concentration-dependent inhibition of protein denaturation. Among them, compound **3** demonstrated the highest protective effect, closely approximating the standard drug indomethacin at the highest tested concentration (500 µg/mL). Compound **2** showed moderate inhibition, while compound **1** displayed the least activity. These findings were consistent across both models, indicating that the synthesized derivatives, particularly compound **3**, possess notable protein-stabilizing properties. Although protein denaturation assays do not directly measure anti-inflammatory potency, the results suggest a potential cytoprotective effect that may contribute to the overall anti-inflammatory profile of these compounds. The evaluated compounds’ % inhibition for the bovine serum albumin and egg albumin testing were displayed below (Figure 2A,B).

##### Effect on Erythrocyte Membrane Stability

The synthesized compounds were further evaluated for their membrane-stabilizing activity using the heat-induced hemolysis model in rat red blood cells (RBCs). All three compounds exhibited protective effects against hemolysis in a concentration-dependent manner. At a concentration of 400 µg/mL, compound 1 inhibited hemolysis by 94.6%, compound 2 by 93.9%, and compound 3 by 95.2%, compared to 94.5% inhibition by indomethacin. These results indicate that the compounds provided a level of protection comparable to the standard drug, without significant deviation. Therefore, the membrane stabilization effects of the synthesized derivatives may contribute to their potential anti-inflammatory activity by preventing lysosomal membrane damage under hypotonic stress (Figure 2C).

##### In Vitro COX Inhibitory Assay

**Figure 3:**
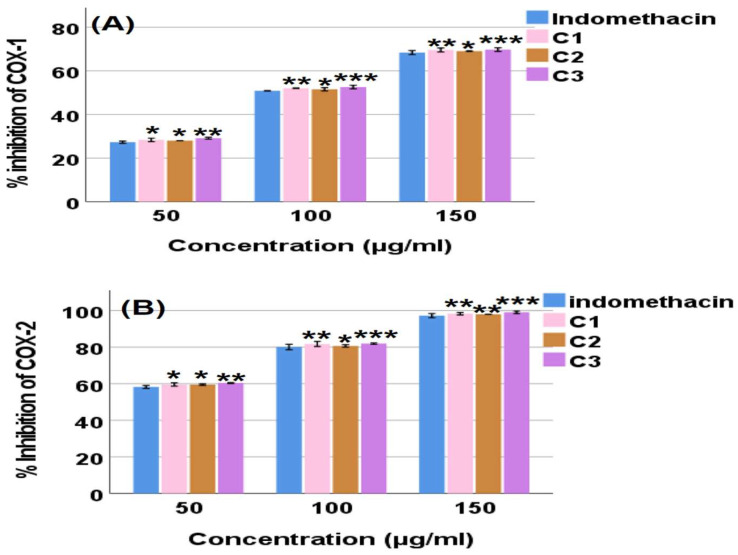
Effect of compounds **1**, **2**, and **3** on (**A**) cyclooxygenase-1 inhibition and (**B**) cyclooxygenase-2 inhibition by in vitro study. All values are expressed as mean ± SD (n = 3), using one-way ANOVA followed by Tukey’s posttest. C1, C2, C3: compound **1**–**3**. * *p* ≤ 0.05 (significant), ** *p* ≤ 0.01 (very significant), *** *p* ≤ 0.001 (highly significant) vs. Indomethacin.

Figure 3 displays a summary of the COX inhibition results utilizing compounds **1**, **2**, and **3**. The mean of the COX inhibition activity in the three compounds, according to the test, was used to determine the average percentage of COX-1 and COX-2 inhibition. In comparison to indomethacin (mean activity COX-1, 68%, and COX-2, 97%), it was found that compound 1 (mean activity COX-1, 70%, and COX-2, 98%), compound 2 (mean activity COX-1, 69%, and COX-2, 98%), and compound 3 (mean activity COX-1, 70%, and COX-2, 99%) were significant inhibitors of COX-1 and 2.

##### Cell Cytotoxicity

**Figure 4:**
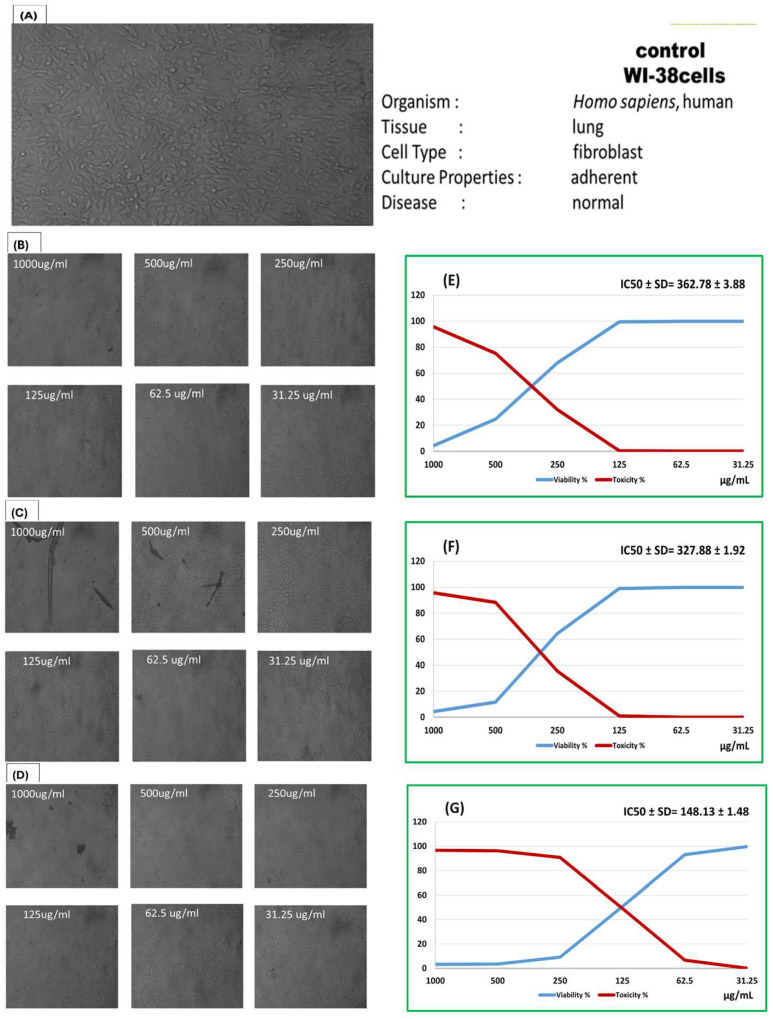
Representative micrographs of in vitro cell cytotoxicity assessment of different concentrations of compounds **1**–**3** on WI-38 human lung fibroblast cells. (**A**) Control image showing untreated WI-38 cells with typical fibroblast morphology (elongated and spindle-shaped). (**B**–**D**) Morphological changes in WI-38 cells treated with increasing concentrations of compound **1**–**3** (31.25 to 1000 µg/mL). (**E**) Dose–response curve for compound **1**, showing cell viability (blue line) and toxicity (red line). IC_50_ = 362.78 ± 3.88 µg/mL. (**F**) Dose–response curve for compound **2**. IC_50_ = 327.88 ± 1.92 µg/mL. (**G**) Dose–response curve for compound **3**. IC_50_ = 148.13 ± 1.48 µg/mL. **Note**: Cell viability and toxicity were determined using a standard MTT assay. A dose-dependent decrease in viability and increase in toxicity were observed for all compounds, with compound **3** showing the highest cytotoxicity based on the lowest IC_50_ value.

We evaluated the cytotoxic effect of the compounds **1**, **2**, and **3** on the Wi-38 cells using the MTT test. We treated the cell line with tested compounds in concentrations ranging from 31.25 to 1000 µg/mL and calculated the half-maximal inhibitory concentration (IC_50_) of **1**, **2**, and **3** tested compounds; the IC_50_ values were 362.78, 327.88, and 148.13 µg/mL, respectively. All tested compounds showed the highest percentage of cell viability (99.9%) for compounds **1** and **2** and 99.8% for compound **3** at a concentration of 31.25 µg/mL, indicating the least cytotoxicity. Additionally, we analyzed the cell morphological changes in cultures before and after exposure to the tested compounds (Figure 4) and found morphology changes in a dose-dependent manner. Cells start to shrink and lose their fibroblast-like structures in concentrations above 125 µg/mL for compounds **1** and **2** and above 62.5 µg/mL for compound **3**. Compounds **1** and **2** were demonstrated to have lower toxicity than compound **3**, indicating that compounds **1** and **2** are worthy of further evaluation.

#### 3.2.2. In Vivo Anti-Inflammatory Activities

##### Acute Toxicity Study

Since no mortality or severe toxicity signs were observed at an intraperitoneal dose of 200 mg/kg (equivalent to one-tenth of the estimated LD_50_), compounds **1**, **2**, and **3** demonstrated a generally safe profile, although mild diarrhea was noted in all treated groups. Table 1 summarizes the behavioral alterations at this dosage.

##### Effect on Carrageenan-Induced Edema

**Figure 5:**
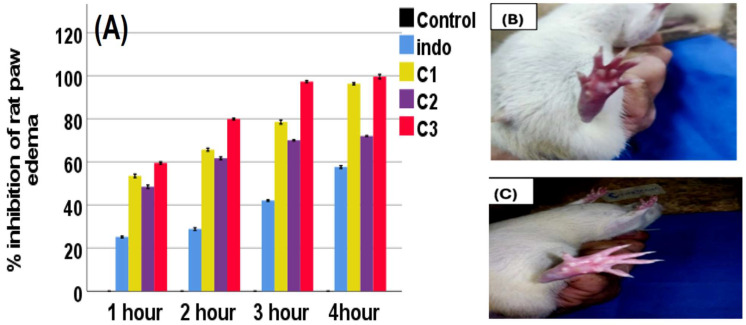
(**A**) Representative photo showing edema inhibition activity of compounds **1**, **2**, and **3** after **1**, **2**, **3**, and **4** h from the carrageenan injection, which caused swelling and redness of the injected paw (**B**,**C**). Indo: indomethacin; C1, C2, C3: compound **1**–**3**.

Rats in the Carr group, who received a 1% carrageenan injection, had redness, hotness, and swelling in their left hind paw, as seen in Figure 5. The paw edema volume also significantly increased after 1, 2, 3, and 4 h postinjection. However, both before and after the carrageenan injection, the groups treated with indomethacin and other compounds showed an improvement in the volume of edema at all periods compared to the carrageenan group. The studied compounds **1**, **2**, and **3** demonstrated marked anti-inflammatory activity at 4 h with percentage inhibition of 96.31%, 72.08%, and 99.69%, respectively. These values indicate a greater improvement compared to the reference drug indomethacin, which showed 57.66% inhibition. After three and four hours, the target compounds’ anti-inflammatory activity was significantly enhanced. During all periods, compounds **1** and **3** exhibited the greatest suppression of paw edema (respectively, 53.6% and 59.5% after 1 h, 65.74% and 79.93% after 2 h, 78.52% and 97.23% after 3 h, and 96.31% and 99.69% after 4 h). More potent than indomethacin, compound **3** displayed a synergistic anti-inflammatory effect.

##### Inflammatory Cytokine Changes

The carrageenan-induced inflammation group exhibited a significant elevation in serum levels of pro-inflammatory markers compared to the control group, with measured concentrations of TNF-α (13.90 ± 0.34 pg/mL), IL-1α (105.72 ± 0.73 pg/mL), IL-1β (150.16 ± 0.60 pg/mL), IL-6 (21.08 ± 0.51 pg/mL), and CRP (10.04 ± 0.70 pg/mL). These values reflect the marked systemic inflammatory response elicited by carrageenan injection. Treatment with the synthesized compounds (**1**, **2**, and **3**) resulted in a significant reduction in the elevated cytokine and CRP levels when compared to the carrageenan group. Among the tested compounds, compound **3** exhibited the most pronounced effect, restoring the measured parameters to values closely comparable with those of the normal control group. Compound **1** also showed substantial anti-inflammatory activity, particularly in lowering IL-1β and CRP levels. In contrast, compound **2** showed a moderate but statistically significant decrease in all tested markers. When compared to the reference drug indomethacin, all three compounds showed greater reduction in TNF-α, IL-1β, and CRP levels, indicating a potentially stronger anti-inflammatory effect, particularly with compound **3**, which demonstrated the highest efficacy among the tested derivatives (Table 2).

##### Oxidative Stress Biomarker Changes

**Figure 6:**
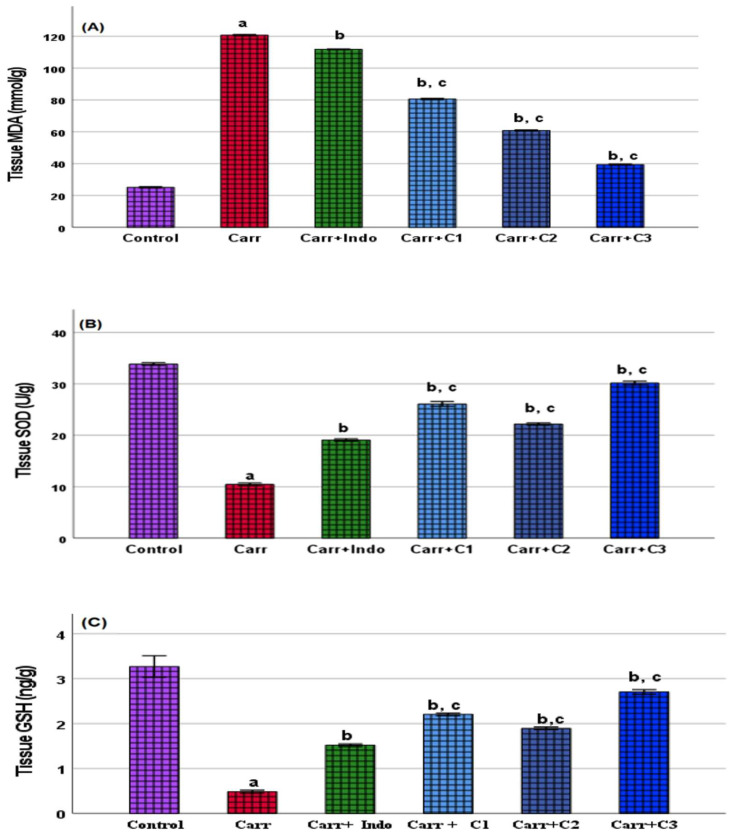
Paw tissue oxidative damage biomarkers of rats in groups (**A**) MDA, (**B**) SOD, and (**C**) GSH. Each value represents the mean ± SD for 6 rats per group. Results are considered significantly different when *p* < 0.05. ^a^ significant difference compared to the control. ^b^ significant difference compared to the carrageenan group. ^c^ significant difference compared to the Indo group. Carr: carrageenan; Indo: indomethacin; MDA: malondialdehyde; SOD: superoxide dismutase; GSH: reduced glutathione.

Figure 6A–C illustrates the impact of carrageenan injection on oxidative stress markers in rat paw tissue. The carrageenan group showed a marked increase in malondialdehyde (MDA) levels (~122 nmol/g), representing a more than 4.5-fold elevation compared to the control group (~27 nmol/g), indicating extensive lipid peroxidation. Additionally, superoxide dismutase (SOD) activity was significantly reduced by approximately 60%, and glutathione (GSH) levels dropped by more than 85% relative to the control. Treatment with the synthesized compounds significantly ameliorated these alterations. Compound **3** exhibited the strongest antioxidant effect, reducing MDA levels close to normal and restoring both SOD activity and GSH concentration to near-control values. Compounds **1** and **2** also demonstrated notable improvements, but to a lesser extent than compound **3**. Notably, all three compounds outperformed indomethacin in restoring oxidative balance, suggesting superior antioxidant and cytoprotective capacities.

##### COX Inhibition Assay

**Figure 7:**
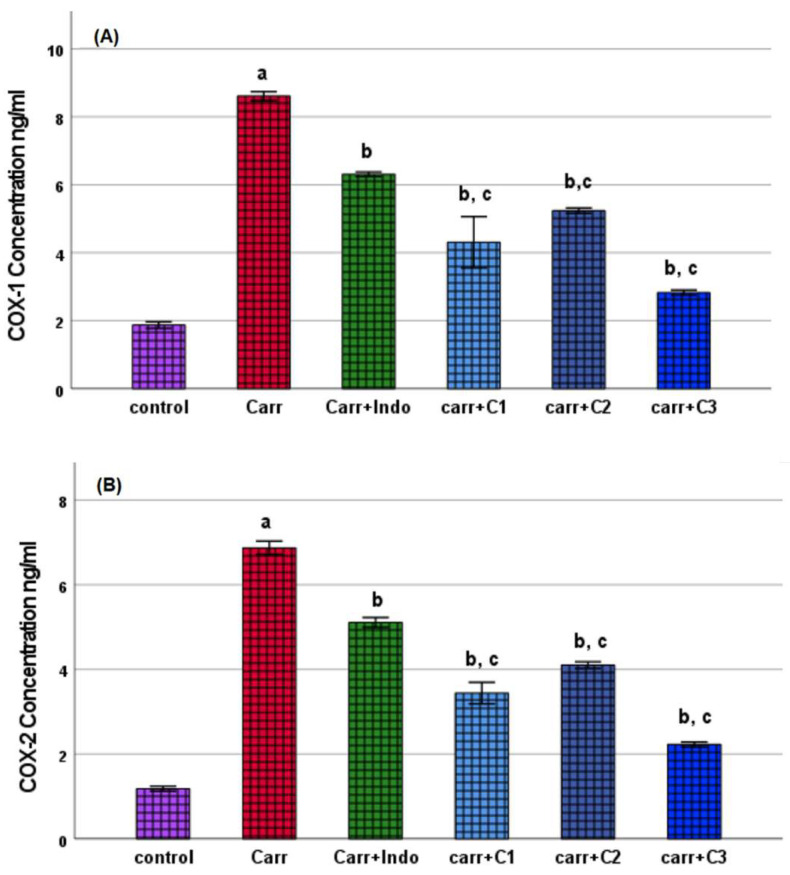
Compounds 1, 2, and 3 reduced significantly (**A**) COX-1 and (**B**) COX-2 levels in the carrageenan-injected paw. Each value is the mean ± SD for 6 rats per group. Above each column, different letters mean statistical significance at (*p* < 0.05). ^a^ significant difference compared to the control. ^b^ significant difference compared to the carrageenan group. ^c^ significant difference compared to the Indo group. Carr: carrageenan; Indo: indomethacin; COX: cyclooxygenase.

Both COX-1 and COX-2 were significantly elevated in the group that received carrageenan administration, as shown in Figure 7A,B. Compounds **1**, **2**, and **3** produced significant reductions in both COX-1 and COX-2 levels, as demonstrated by the following: COX-1, 4.31 ± 0.90 ng/mL and COX-2, 3.44 ± 0.30 ng/mL for compound **1**; COX-1, 5.24 ± 0.09 ng/mL and COX-2, 4.10 ± 0.09 ng/mL for compound **2**; and COX-1, 2.82 ± 0.08 ng/mL and COX-2, 2.22 ± 0.06 ng/mL for compound **3**. Both COX-1 and COX-2 concentrations are lowered by the standard drug indomethacin, reaching 6.31 ± 0.08 ng/mL and 5.11 ± 0.13 ng/mL, respectively. From our mentioned results, the three compounds outperformed the standard drug in terms of effect by a significantly high percentage, especially compound **3**.

##### Assessment of Therapeutic Index and COX Inhibitory Profiles

Table 3 indicates that three test compounds were evaluated for their COX-2 and COX-1 inhibitory activities, cytotoxicity (IC_50_), and therapeutic index (TI). Compound **1** exhibited the highest IC_50_ value (362.78 µg/mL), indicating the lowest cytotoxicity, along with strong COX-2 inhibition (98%) and moderate COX-1 inhibition (70%), resulting in the highest TI (~7.4). Compound **2** showed similar COX-2 inhibition (98%) and slightly lower COX-1 selectivity (69%) with an IC_50_ of 327.88 µg/mL and a TI of ~6.6. Compound **3** demonstrated the highest COX-2 inhibition (99%) but also the lowest IC_50_ (148.13 µg/mL), reflecting greater cytotoxicity and a narrower TI (~2.9). These results suggest that compound **1** has the most favorable safety profile, while compound **3**, despite its high efficacy, may require dose optimization to mitigate cytotoxic effects.

##### Histopathological Assessment of Paw Tissue

**Figure 8:**
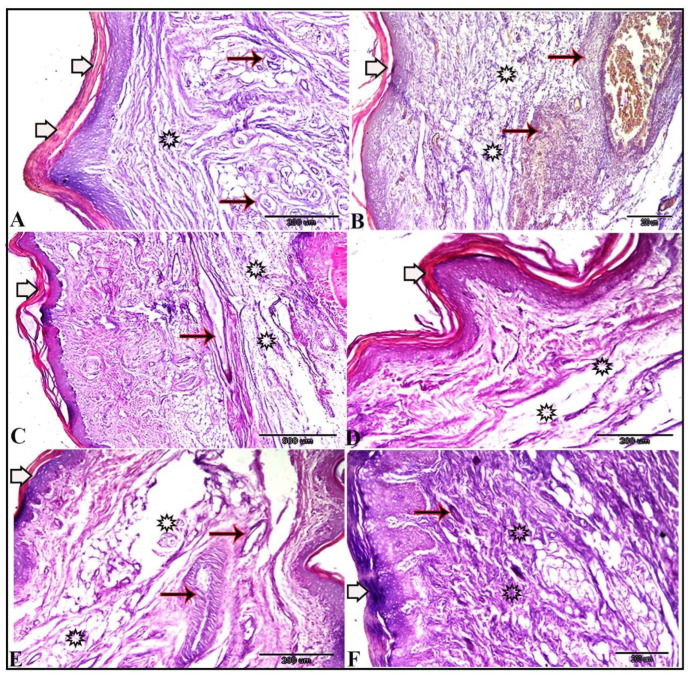
Photomicrograph of paw tissue of rats from experimental groups stained with HE: (**A**) The normal control group shows the normal appearance of the epidermis (white arrowheads) and dermis without any lesion (star) and normal dermal vessels (arrows). (**B**) Carrageenan-injected paw tissue shows a thin epidermis (white arrow), massive edema (stars), and marked vasodilation with severe inflammatory cellular infiltration (arrow). (**C**) The carrageenan-injected paw of rats treated with indomethacin shows a thin epidermis (white arrow), marked edema (stars), and dilated and congested dermal vessels (arrow). (**D**) The carrageenan-injected paw of rats treated with compound **1** shows a normal epidermis (white arrow) and mild edema (stars). (**E**) The carrageenan-injected paw of rats treated with compound **2** shows more or less epidermal thickness (white arrow), moderate edema (stars), and more or less normal dermal vessels (arrow). (**F**) The carrageenan-injected paw of rats treated with compound **3** shows normal epidermal thickness (white arrow), normal dermis (stars), and normal dermal vessels (arrow).

**Figure 9:**
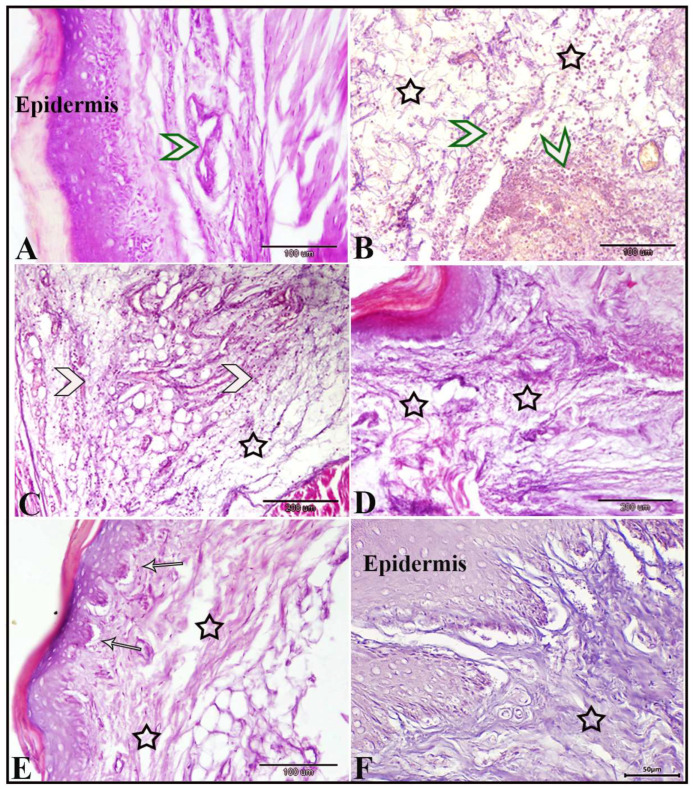
Photomicrograph of paw tissue of rats from experimental groups stained with HE: (**A**) The normal control group shows the normal appearance of the epidermis and dermis without any lesion, with normal dermal vessels (arrows). (**B**) The carrageenan-injected paw tissue shows massive edema (stars) and marked inflammatory cellular infiltration (arrowheads). (**C**) The carrageenan-injected paw of rats treated with indomethacin shows marked edema (stars) and inflammatory cellular infiltration (arrowheads). (**D**) The carrageenan-injected paw of rats treated with compound **1** shows a mild inflammatory dermal edema (stars). (**E**) The carrageenan-injected paw of rats treated with compound **2** shows moderate edema (stars), mild dermal vessels, and inflammatory cellular infiltration (arrows). (**F**) The carrageenan-injected paw of rats treated with compound **3** shows normal epidermal thickness and normal dermis without any lesion (star).

The histopathological examination of paw tissues from different experimental groups showed that the paw tissues from the normal control group exhibited normal histological architecture; the epidermis appeared intact with normal thickness stratified squamous epithelium cornified, and normal dermal structure. The dermis showed no evidence of any lesions with dermal vessels (Figure 8 and Figure 9A). Carrageenan-injected group paw tissues displayed significant pathological changes. The epidermis was noticeably thin, and the dermis exhibited massive edema. Additionally, the dermal vessels showed marked vasodilation accompanied by severe inflammatory cellular infiltration (Figure 8 and Figure 9B). In carrageenan-injected rats treated with indomethacin, the epidermis remained thin, and the dermis exhibited marked edema. Furthermore, the dermal vessels were dilated and congested, with evident inflammatory cellular infiltration (Figure 8 and Figure 9C). Treatment with compound **1** ameliorated some of the pathological changes induced by carrageenan. The epidermis appeared normal, while the dermis displayed only mild edema (Figure 8 and Figure 9D). Rats treated with compound **2** showed a partially improved histological structure. The epidermis exhibited nearly normal thickness, and the dermis demonstrated moderate edema. Mild inflammatory infiltration was observed around dermal vessels (Figure 8 and Figure 9E). Treatment with compound **3** resulted in the most pronounced protective effects against carrageenan-induced damage. The epidermis appeared normal in thickness, and the dermis exhibited a normal histological structure without edema or lesions. The dermal vessels were also normal, with no evidence of congestion or inflammatory infiltration (Figure 8 and Figure 9F). These findings indicate that compound **3** exhibited superior anti-inflammatory and tissue-protective effects compared to compound **1**, compound **2**, and indomethacin. Compound **3** restored the normal histological architecture of the paw tissue, suggesting its potential as a therapeutic agent for inflammation.

**Figure 10:**
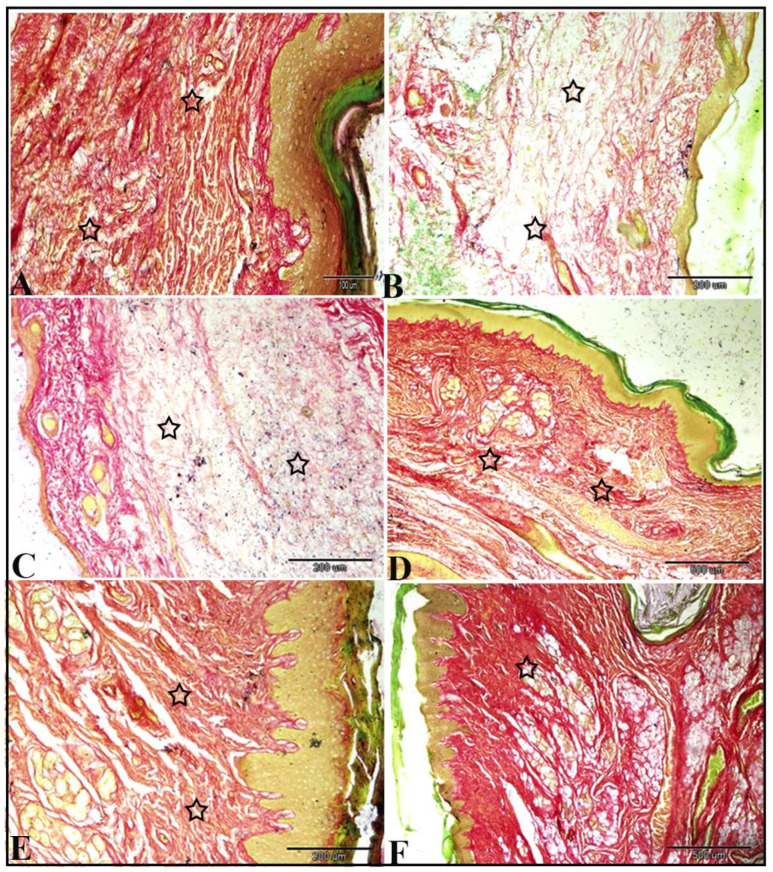
Photomicrograph of paw tissue of rats from experimental groups stained with Sirius Red stain: (**A**) The normal control group shows the normal appearance of dermal collagen (stars). (**B**) The carrageenan-injected paw tissue shows few dispersed collagenous fibers in the edematous dermis (stars). (**C**) The carrageenan-injected paw of rats treated with indomethacin shows marked dispersed collagenous fibers (stars). (**D**) The carrageenan-injected paw of rats treated with compound **1** shows normal collagen quantity and distribution (stars). (**E**) The carrageenan-injected paw of rats treated with compound 2 shows normal dermal collagen (stars). (**F**) The carrageenan-injected paw of rats treated with compound 3 shows normal dermal collagenous fibers without any lesion (star).

The collagen content and distribution in the dermis of paw tissues from different experimental groups, stained with Sirius Red, are presented in Figure 10. The dermal collagen in the normal control group appeared well-organized and evenly distributed. The collagen fibers maintained a normal quantity and structure throughout the dermis (Figure 10A). Carrageenan-injected group paw tissues from the carrageenan-injected group displayed significant disruption in collagen organization. The dermis contained few and sparsely distributed collagenous fibers, which were dispersed within the edematous tissue (Figure 10B). In carrageenan-injected rats treated with indomethacin, the collagen fibers were markedly dispersed and less organized within the dermis. The disruption in collagen structure was still evident (Figure 10C). Treatment with compound **1** improved collagen distribution in the dermis. The collagen fibers appeared normal in both quantity and organization, indicating partial restoration of the dermal structure (Figure 10D). Rats treated with compound **2** showed an even better restoration of dermal collagen. The collagen fibers displayed a normal appearance and distribution, resembling the structure observed in the normal control group (Figure 10E). Compound **3**-treated group (Figure 10F): Treatment with compound **3** resulted in a complete restoration of normal dermal collagen. The collagen fibers were well organized and evenly distributed without any signs of disruption or lesion (Figure 10F). The Sirius Red staining revealed significant disruption of collagen fibers in the dermis of carrageenan-injected rats, with partial restoration observed in the indomethacin and compound **1** treatment groups. Compound **3** demonstrated the most pronounced protective effect, restoring the normal quantity and distribution of dermal collagen, indicating its potential role in preserving tissue integrity during inflammation.

##### Histomorphometry Analysis

**Figure 11:**
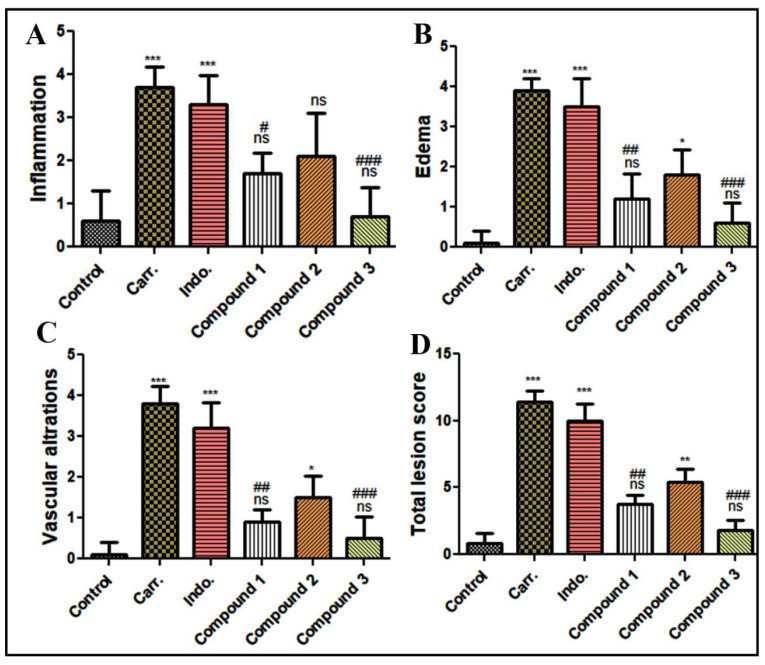
Histomorphometry graph showing semiquantitative measurements of lesion scores recorded in PAW tissue sections among the experimental groups. (**A**) Inflammation, (**B**) edema, (**C**) vascular alterations, (**D**) total lesion scores. Data are expressed as means ± standard deviations. Significant differences vs. the control group are marked by different asterisks, and significant differences vs. Carr. The treated group is marked by different # through the Kruskal–Wallis test with Dunn’s Multiple Comparison post hoc test: (*^,#^ *p* ≤ 0.05, **^,##^ *p* ≤ 0.01, ***^,###^ *p* ≤ 0.001), ns refers to non-significant. Carr: carrageenan; Indo: indomethacin.

The histomorphometry evaluation of lesion scores in paw tissue sections, including inflammation, edema, vascular alterations, and total lesion scores, is summarized in Figure 11. The inflammation scores significantly increased in the carrageenan-injected group compared to the normal control group (*p* ≤ 0.001). Treatment with indomethacin, compound **1**, and compound **3** significantly reduced inflammation scores compared to the carrageenan group (*p* ≤ 0.05, *p* ≤ 0.001, respectively). Compound **3** demonstrated the most notable reduction, with inflammation scores approaching those of the normal control group. Edema scores were markedly elevated in the carrageenan-injected group compared to the normal control group (*p* ≤ 0.001). Treatment with the tested compounds significantly decreased edema scores. Among the treatments, compound **3** exhibited the greatest efficacy, leading to edema scores comparable to those of the control group. Vascular alteration scores were significantly higher in the carrageenan-injected group relative to the control group (*p* ≤ 0.001). Treatment with indomethacin and the test compounds significantly mitigated vascular alterations. Compound **3** showed the most pronounced effect, with vascular alteration scores returning to near-normal levels. The total lesion scores, reflecting the combined severity of inflammation, edema, and vascular alterations, were significantly increased in the carrageenan-injected group (*p* ≤ 0.001) compared to the control group. Treatment with indomethacin and the test compounds reduced total lesion scores significantly in Compounds **1** and **3** (*p* ≤ 0.01 and *p* ≤ 0.001, respectively). Compound **3** again exhibited the highest efficacy in restoring total lesion scores to levels comparable to the normal control group. The histomorphometric analysis revealed that carrageenan injection caused significant pathological changes in paw tissue, as indicated by increased lesion scores across all evaluated parameters. Treatment with indomethacin and the tested compounds effectively mitigated these changes, with compound **3** showing the most pronounced protective and restorative effects. These findings highlight the potential of compound **3** as a therapeutic agent for managing inflammation-induced tissue damage.

## 4. Discussion

The promising in vitro anti-inflammatory effects of compounds **7**, **8**, and **12**, previously reported in our earlier study [23], served as a foundation for the current research. Building upon these findings, the present study aimed to further assess the biological activity and cytotoxicity of the structurally related synthesized compounds (**1**, **2**, and **3**) through a comprehensive set of in vitro and in vivo assays. These included protein denaturation, erythrocyte membrane stabilization, COX enzyme inhibition, and MTT-based cytotoxicity assays. The extension of the evaluation to in vivo models was essential to confirm and deepen our understanding of the anti-inflammatory potential of these compounds and their biocompatibility profiles.

Protein denaturation is a well-documented mechanism of inflammation in which the tertiary and secondary structures of proteins are disrupted under stress, leading to the formation of autoantigens [43]. This process is associated with several chronic inflammatory diseases, including rheumatoid arthritis and lupus erythematosus [44]. In the current investigation, there was a notable (*p* < 0.05) reduction in protein denaturation of both bovine serum albumin and egg albumin in the presence of all three tested compounds, as well as the standard drug, indomethacin. Compared to indomethacin, compounds **1**, **2**, and **3** demonstrated a superior percentage inhibition of protein denaturation at concentrations ranging from 50 to 1000 µg/mL. These findings suggest that the tested compounds may help regulate the production of autoantigens by preventing protein denaturation in inflammatory conditions. These results are consistent with previous reports demonstrating that inhibition of protein denaturation is a useful indicator of anti-arthritic activity [45,46].

Although the protein denaturation assay is widely used in preliminary anti-inflammatory screening, it is a non-specific model that may reflect chaperone-like stabilization of proteins rather than true anti-inflammatory action [47]. The mechanism by which indomethacin inhibits denaturation is not fully understood and may involve non-specific hydrophobic interactions [48]. Therefore, its use here is comparative, not mechanistic. Future studies could include chemical chaperones like ursodeoxycholic acid or sodium 4-phenylbutyrate to better differentiate protein stabilization from anti-inflammatory activity. Thus, while useful, this assay was supported by additional specific models such as COX inhibition and cytokine analysis.

Stabilization of lysosomal and erythrocyte membranes is another hallmark of anti-inflammatory agents, as it prevents the release of inflammatory mediators such as histamines and proteases [49]. The ability of compounds **1**, **2**, and **3** to prevent hemolysis of rat RBCs in hypotonic solution showed strong membrane-protective effects, comparable or superior to indomethacin, a standard NSAID. At 400 µg/mL, compound **3** showed the highest inhibition (95.2%), slightly higher than indomethacin (94.5%). These results suggest that compound **3** may act by inhibiting the lysis of biological membranes, a known mechanism of anti-inflammatory agents.

Our findings are consistent with previous research assessing both synthetic and natural anti-inflammatory drugs. For example, flavonoid and polyphenol investigations showed similar inhibitory effects on RBC lysis and protein denaturation, indicating that our compounds may have similar mechanisms of action [50,51,52].

Cyclooxygenase enzymes (COX-1 and COX-2) are key players in the conversion of arachidonic acid to prostaglandins, which are mediators of inflammation and pain [53]. The inhibition of COX, particularly COX-2, is a critical mechanism of action of most NSAIDs [53]. Our study showed that all three compounds inhibited COX activity in a dose-dependent manner, as measured by the colorimetric assay based on TMPD oxidation. This provides evidence for the concept that COX inhibition may be partially responsible for the tested compounds’ anti-inflammatory effects. The compounds’ efficacy was further confirmed by our study’s use of indomethacin as a reference.

Evaluation of cytotoxicity on the Wi38 cell line revealed that compounds **1** and **2** had relatively low toxicity, with IC_50_ values of 362.78 and 327.88 µg/mL, respectively, compared to compound **3** (IC_50_ = 148.13 µg/mL). The high percentage of cell viability at lower concentrations indicates the compounds’ potential safety at therapeutic doses. Morphological assessments confirmed that compound **3** induced cytotoxic changes at lower concentrations than compounds **1** and **2**, suggesting a narrower therapeutic window. These findings are in line with other studies highlighting the importance of balancing anti-inflammatory efficacy with cytotoxic safety when developing new therapeutic agents [54,55]. Our in vitro anti-inflammatory assays demonstrated that all three compounds possess promising biological activity, with compound **3** being the most potent in terms of anti-inflammatory effect but with higher cytotoxicity. Compounds **1** and **2** showed a more favorable balance between efficacy and safety, making them suitable candidates for in vivo evaluation.

Although our cytotoxicity assessment provided a useful initial indication of safety, future studies will include a broader panel of normal and target-specific cell lines as well as detailed histological examination of vital organs to comprehensively characterize the toxicological profile of the tested compounds.

Consequently, we have determined the therapeutic index (TI) for compounds **1**, **2**, and **3** as the ratio of the dose/concentration corresponding to COX inhibition to the IC_50_ (derived from the MTT cytotoxicity assay). The ratio of a compound’s toxicity (IC_50_) to its effective dose (e.g., COX inhibition concentration) is known as the TI. Since the drug is effective at a dose well below its dangerous level, a higher TI denotes a greater safety margin. Because the toxic and effective dosages are closer together, a lower TI indicates a smaller safety buffer, which raises concerns about potential adverse effects [56]. Strong anti-inflammatory effects with minimal cytotoxicity are indicated by the excellent therapeutic indices of compounds **1** and **2**. They represent great prospects for further development. Despite being the most effective in reducing COX-2 and inflammation, compound **3** has a lower TI, which suggests a higher risk of cytotoxicity. Although structural changes would be required to lower toxicity or enhance selectivity, it might be used as a pioneer molecule.

As mentioned in the introduction, Ferrer et al. (2019) highlight the significance of creating COX-2-specific inhibitors to reduce gastrointestinal adverse effects linked to non-selective COX inhibition [2]. In vitro, the synthetic compounds **1**, **2**, and **3** showed significant inhibition of both COX-1 and COX-2; however, their broader anti-inflammatory mechanisms, including antioxidant activity (SOD, GSH elevation, MDA reduction) and cytokine suppression (TNF-α, IL-1β, IL-6), suggest a multi-targeted therapeutic profile [57].

Since the compounds’ superior safety profile (no toxicity at 200 mg/kg) and efficacy (e.g., compound **3**’s 99.69% paw edema inhibition) might exceed COX-1-related concerns, this dual inhibition may not necessarily conflict with the goal of selectivity. Interestingly, the COX-2 inhibition of compound **3** (99%) significantly surpassed that of COX-1 (70%), suggesting partial selectivity (Figure 3). While maintaining the reported pleiotropic advantages, more structural playing directed by docking studies may improve COX-2 specificity [9,58].

The acute toxicity assessment revealed no mortality or significant behavioral alterations at an intraperitoneal dose of 200 mg/kg, indicating that all tested compounds possess a high safety margin. According to the OECD guidelines for testing of chemicals, the absence of lethality at doses up to 2000 mg/kg body weight generally classifies a compound as practically non-toxic [59]. This supports us in vivo pharmacological testing and suggests a low risk of systemic toxicity for the studied agents.

The carrageenan-induced paw edema model is a classical and widely accepted method for evaluating the acute phase of inflammation, which is biphasic. The early phase (0–2 h) is mediated by histamine, serotonin, and bradykinin, while the late phase (3–5 h) is attributed to prostaglandins and pro-inflammatory cytokines such as TNF-α and IL-1β [60,61].

Our findings revealed that all test compounds, particularly compound **3**, significantly inhibited paw edema during both the early and late phases. The maximum inhibition was observed at the 4 h mark, with compounds **1** and **3** demonstrating superior anti-inflammatory efficacy (96.31% and 99.69%, respectively) compared to the standard drug, indomethacin (57.66%). These results indicate that the test compounds possess both antihistaminic and anti-prostaglandin properties, contributing to their strong anti-edematous effects. Compound **3**’s superior performance suggests a possible synergistic or multi-target mechanism that may involve both COX inhibition and modulation of inflammatory mediators, consistent with in vitro COX data. This may position compound **3** as a potential lead compound for further development.

Inflammatory cytokines play a central role in propagating and maintaining the inflammatory response. Elevated serum levels of TNF-α, IL-1α, IL-1β, IL-6, and CRP in the carrageenan group confirm the systemic inflammatory response. Treatment with compounds **1**, **2**, and **3** significantly reduced the levels of these cytokines, restoring them to near-normal values. This indicates that the compounds may modulate the immune response by suppressing key inflammatory signaling pathways such as NF-κB and MAPK, both of which are upstream regulators of cytokine expression [62,63].

Interestingly, all three tested compounds suppressed cytokine levels better than indomethacin. This indicates that their anti-inflammatory properties include more extensive immunomodulatory actions in addition to COX inhibition, which is especially helpful in the treatment of inflammatory illnesses requiring immunological dysregulation.

Oxidative stress is a hallmark of acute and chronic inflammation. Carrageenan-induced inflammation elevates reactive oxygen species (ROS), leading to increased lipid peroxidation (measured as MDA) and depletion of endogenous antioxidants like SOD and GSH [64,65].

Our study revealed a marked decrease in SOD and GSH levels in the inflamed tissue, along with a significant rise in MDA in the carrageenan group. Treatment with compounds **1**, **2**, and **3** reversed these effects, suggesting potent antioxidant activity. The elevation of SOD and GSH levels and suppression of MDA suggest that the tested compounds reduce oxidative stress, either through direct radical scavenging or by upregulating endogenous antioxidant defenses.

Because oxidative stress is closely related to the inflammatory response and tissue damage, this antioxidant capability and cytokine regulation greatly contribute to the observed anti-inflammatory effects [66,67].

The observed anti-inflammatory effects of Compounds **1**–**3** may be attributed to their potential influence on key signaling pathways such as NF-κB and MAPK, which are widely known to regulate inflammatory responses. However, since these pathways were not directly investigated in this study, further mechanistic analyses are required to confirm their involvement.

Nonetheless, numerous studies have demonstrated that 1,3,5-triazine and triazolotriazine derivatives possess potent anti-inflammatory activity, often attributed to their ability to modulate key signaling pathways such as NF-κB and MAPK. In particular, these scaffolds have been reported to suppress the expression of pro-inflammatory cytokines (e.g., TNF-α, IL-1β, IL-6) and downregulate COX-2, thus disrupting the NF-κB/COX-2 positive feedback loop. The sulfonamide moiety, also present in our compounds, is a well-known pharmacophore involved in COX enzyme inhibition. Therefore, based on these established structure–activity relationships, it is plausible that our compounds exert their effects through similar molecular mechanisms [9,12,13].

Notably, treatment with compound **3** resulted in a significant reduction in pro-inflammatory cytokines, including TNF-α (7.16 ± 0.13 pg/mL vs. 13.90 ± 0.34 in the carrageenan group) and IL-6 (11.39 ± 0.23 pg/mL vs. 21.08 ± 0.51). This pattern suggests effective suppression of NF-κB activation. In addition, COX-2 expression was markedly downregulated following treatment with compound **3** (2.22 ± 0.06 ng/mL vs. 15.11 ± 0.13 in the carrageenan group), indicating a possible interruption of the NF-κB/COX-2 positive feedback loop.

A pronounced decrease in lipid peroxidation markers such as MDA (35.71 ± 0.51 vs. 121.71 ± 0.51 nmol/g tissue) suggests suppression of ROS-induced activation of the p38 and JNK pathways. IL-1β levels were normalized (60.38 ± 0.73 vs. 150.16 ± 0.60 pg/mL), indicating effective inhibition of MAPK-mediated cytokine production. A significant enhancement of antioxidant enzyme SOD (38.48 ± 0.31 vs. 10.48 ± 0.31 U/mg protein) further supports interference with MAPK phosphorylation events.

The anti-inflammatory efficacy of the synthesized compounds was further confirmed through detailed histopathological and histomorphometry assessments. Carrageenan-induced paw tissue damage was characterized by dermal edema, vascular congestion, and significant inflammatory cell infiltration. Treatment with compounds **1**, **2**, and **3** substantially ameliorated these changes, with compound **3** showing the most profound protective effect, restoring normal epidermal and dermal architecture and eliminating signs of inflammation. In addition, Sirius Red staining was employed to assess collagen integrity, revealing marked collagen fiber disorganization in the carrageenan group. Treatment with the test compounds, especially compound **3**, restored the normal density and orientation of dermal collagen fibers, closely resembling the control group’s structural integrity. These findings collectively confirm that the compounds not only reduce inflammatory markers but also preserve tissue structure, supporting their potential as effective therapeutic agents for inflammatory conditions.

Despite the promising anti-inflammatory and antioxidant effects observed in this study, several limitations should be acknowledged. First, while the compounds demonstrated significant COX-1 and COX-2 inhibition, molecular docking or crystallographic analyses were not performed to elucidate binding interactions or explain the observed lack of selectivity. Second, the compounds exhibited limited COX-2 selectivity, which may pose a risk of gastrointestinal side effects typical of non-selective NSAIDs. Third, the in vivo model employed—carrageenan-induced paw edema—reflects acute inflammation; thus, further studies using chronic inflammation models such as adjuvant-induced arthritis are warranted to assess long-term efficacy. Fourth, although compound **3** showed superior anti-inflammatory activity, its relatively narrow therapeutic index (TI ≈ 2.9) raises concerns regarding cytotoxicity, suggesting a need for structural optimization. Lastly, no pharmacokinetic parameters (e.g., absorption, distribution, metabolism, or excretion) were evaluated, which are critical for clinical translation and should be addressed in future work.

The in vitro anti-inflammatory activities of the synthesized compounds, as demonstrated by RBC membrane stabilization and COX inhibition assays, showed a consistent correlation with their in vivo efficacy in a carrageenan-induced paw edema model. Notably, compound 3 exhibited the highest in vitro potency and correspondingly produced the greatest in vivo anti-inflammatory response. This correlation was further supported by reductions in pro-inflammatory cytokines, oxidative stress markers, and histopathological improvements, highlighting the predictive value of the in vitro assays for in vivo therapeutic outcomes.

## 5. Conclusions

In summary, the synthesized 1,3,5-triazine-based benzene sulfonamide derivatives demonstrated potent anti-inflammatory activity, both in vitro and in vivo. The compounds effectively inhibited COX-1 and COX-2 enzymes and reduced key pro-inflammatory cytokines (TNF-α, IL-1β, IL-6, CRP) and improved oxidative stress biomarkers by elevating GSH and SOD levels while reducing MDA. Among the tested compounds, compound **3** exhibited the highest anti-inflammatory efficacy, though with relatively higher cytotoxicity, emphasizing the need for further structural optimization to improve its therapeutic index. Histopathological and Sirius Red staining analyses confirmed the protective effects of the compounds on tissue architecture and collagen integrity, particularly with compound **3**. These findings suggest that the tested triazine derivatives, especially compounds **1** and **3**, are promising candidates for further development as anti-inflammatory agents with antioxidant properties. Future studies will focus on pathway-specific analyses and molecular docking to elucidate the exact mechanisms of action and enhance COX-2 selectivity.

## Figures and Tables

**Table 1:** Acute toxicity effect of tested compounds.

Observation	Control	Compound 1	Compound 2	Compound 3
Change in skin color	Normal	Normal	Normal	Normal
Diarrhea	Absent	Present	Present	Present
Seizures	Absent	Absent	Absent	Absent
Micturition	Normal	Normal	Normal	Normal
Drowsiness	Absent	Absent	Absent	Absent
Dyspnea	Absent	Absent	Absent	Absent
Weight loss	No	No	No	No
Ability to feed	Normal	Normal	Normal	Normal
Death	No	No	No	No

**Table 2:** Selective serum inflammatory markers of rats in treated groups compared with the control group.

Groups/ Parameters	CRP (ng/mL)	IL-1α (pg/mL)	IL-1β (pg/mL)	IL-6 (pg/mL)	TNF-α (pg/mL)
Control	1.70 ± 0.05	39.41 ± 0.88	53.25 ± 0.58	10.54 ± 0.24	6.49 ± 0.31
Carr	10.04 ± 0.70 ^a^	105.72 ± 0.73 ^a^	150.16 ± 0.60 ^a^	21.08 ± 0.51 ^a^	13.90 ± 0.34 ^a^
Carr + Indo	6.23 ± 0.22 ^b^	79.28 ± 0.55 ^b^	92.86 ± 0.47 ^b^	18.49 ± 0.27 ^b^	11.30 ± 0.30 ^b^
Carr + C1	4.19 ± 0.28 ^b^	56.97 ± 0.79 ^b^	64.98 ± 0.30 ^b^	14.44 ± 0.26 ^b^	8.27 ± 0.19 ^b^
Carr + C2	3.02 ± 0.28 ^b^	62.82 ± 0.94 ^b^	77.98 ± 0.45 ^b^	16.14 ± 0.16 ^b^	9.38 ± 0.20 ^b^
Carr + C3	2.20 ± 0.36 ^b^	40.66 ± 0.43 ^b^	60.38 ± 0.73 ^b^	11.39 ± 0.23 ^b^	7.16 ± 0.13 ^b^
*p*-value	<0.001	<0.001	<0.001	<0.001	<0.001

Each value represents the mean ± SD. All data having different letters differ significantly at *p* ≤ 0.05. Carr: carrageenan; Indo: indomethacin; TNF–α: tumor necrosis factor-alpha; IL-1: Interleukin-1; IL-6: Interleukin-6; and CRP: C-reactive protein. ^a^ significant difference compared to the control. ^b^ significant difference compared to the carrageenan group.

**Table 3:** Comparative evaluation of COX inhibition, cytotoxicity, and therapeutic index (TI) of test compounds.

Compound	IC_50_ (µg/mL)	COX-2 Inhibition %	COX-1 Inhibition %	COX-2 Inhibition Effective Dose	TI (IC_50_/COX-2 Effective Dose)	Interpretation
1	362.78	98%	70%	49	~7.4	Safest profile. Strong efficacy with minimal cytotoxic risk. Suitable for further development.
2	327.88	98%	69%	49	~6.6	Very good safety margin. Slightly lower than C1 but still acceptable. Promising candidate.
3	148.13	99%	70%	49.5	~2.9	Narrow safety margin. Most efficacious but also most cytotoxic. Further optimization or dose control is needed.

## Data Availability

All data generated or analyzed during this study are included in this published article.

## Abbreviations

NSAIDs	non-steroidal anti-inflammatory drugs
PBS	phosphate-buffered saline
OECD	Organization for Economic Co-operation and Development
ED 50	median effective dose
LD 50	median lethal dose
INF- γ	Interferon γ
NF-κB	nuclear factor kappa B
MAPKs	mitogen-activated protein kinases
COX	cyclooxygenase
RBC	red blood cell
BSA	bovine serum albumin
Indo	indomethacin
Carr	carrageenan
C1, C2, C3	synthesized compounds **1**–**3**
C.F.	chemical formula
Mp.	melting point
M.W	molecular weight
